# Electrolytic Routes to Titanium: Methodological Innovations, Key Findings, and Prospects for Sustainable Production

**DOI:** 10.3390/ma18030525

**Published:** 2025-01-23

**Authors:** Daoguang Du, Zhihe Dou, Tingan Zhang

**Affiliations:** Key Laboratory of Ecological Metallurgy of Multi-Metal Intergrown Ores of Ministry of Education, School of Metallurgy, Northeastern University, Shenyang 110819, China; 2110551@stu.neu.edu.cn

**Keywords:** titanium, electrolysis method, process optimization, electrolysis mechanism, process comparison, future strategy

## Abstract

Titanium is an indispensable strategic metal, and the greening of titanium production processes is a key safeguard for the further development of the titanium industry. Traditional titanium extraction methods involve high-temperature molten salts and high energy consumption, accompanied by significant environmental issues. The electrolytic method for the production of titanium is a more environmentally friendly and promising production process. This review examines recent advancements in electrolytic titanium production, focusing on methods like the FFC, OS, SHS-ED, and USTB processes. These methods offer more efficient and environmentally friendly alternatives to traditional titanium extraction. Key findings include improvements in anode materials, electrolyte compositions, and process optimizations, which enhance titanium purity and production efficiency. The SHS-ED method, in particular, has shown significant advantages by shortening the deoxidation reaction path, improving process efficiency, and reducing the formation of undesirable phases. Despite these advancements, challenges remain in improving current efficiency, reducing energy consumption, and scaling up production. This article aims to provide guidance for future research directions and to discuss how to further promote the development of electrolytic titanium technology for more efficient and environmentally friendly titanium production.

## 1. Introduction

As a rare silver-white transition metal, titanium has melting and boiling points of 1727 °C and 3287 °C, respectively. Titanium has the characteristics of metallic luster, low specific gravity (4.5 g/cm^3^), high melting point, low-temperature resistance, corrosion resistance, and good plasticity. Various elements, such as vanadium and iron, can be melted into high-strength lightweight alloys with titanium in certain proportions, which are widely used in the military, aerospace industry, automobiles, jewelry, kitchen utensils, medicine, sports goods, agricultural food, and other fields [1,2,3]. The utilization value of metal titanium is increasing daily; it is an indispensable strategic metal for improving the quality and level of national defense equipment [4,5]. 

Due to the predominant existence of titanium in the Earth’s crust as refractory oxides, traditional titanium extraction methods, such as the Kroll process and the Hunter process, typically involve high-temperature molten salts and high energy consumption, accompanied by significant environmental issues [6,7]. The Kroll process is currently the predominant method in titanium industry production. In this method, powdered titanium ore (TiO_2_) is placed in a smelting furnace; then, chlorine gas and a reducing agent (coal coke) are introduced, and the temperature is heated to around 1000 °C to achieve a chemical reaction, resulting in the production of liquid titanium tetrachloride [8]. This process generates a large amount of carbon dioxide gas, with the production of one ton of metallic titanium producing approximately 10 tons of carbon dioxide gas. In the magnesium reduction distillation process, a significant amount of electrical energy is consumed, with the production of one ton of sponge titanium requiring the consumption of about 20,000 to 30,000 kilowatt-hours of electricity [9]. Moreover, the removal of impurities in the titanium metal during the distillation reduction process is challenging, leading to high pollution, high energy consumption, and high costs associated with the production of sponge titanium [10]. The Hunter method is another traditional method for titanium extraction. In the first stage, TiCl_4_ and molten sodium react in a reaction vessel to produce TiCl_2_ at approximately 200 °C. The mixture is then transferred to a reaction vessel at 1000 °C with an excess of sodium to complete the reaction [11]. However, in recent years, the Hunter method has been abandoned due to the lack of expansion in equipment capacity and the absence of significant progress in sodium electrolysis production technology. These methods are not only costly but also produce a large number of harmful by-products during the production process, which restricts the sustainable production and application of titanium [9,12,13]. In recent years, with the increasing demands for environmental protection and energy efficiency, researchers have begun to explore greener and more economical titanium extraction technologies [14,15]. Among these emerging technologies, the electrolytic method has garnered widespread attention for its potential to directly extract metallic titanium from titanium oxides under mild conditions [16,17,18].

In the field of electrolytic titanium research, numerous scholars have conducted in-depth reviews and discussions [19,20]. For instance, some studies have explored the advantages and disadvantages of pyrometallurgical and electrometallurgical processes, highlighting the drawbacks of the Kroll and Hunter processes. It has been indicated that with the increasing demand for more environmentally friendly, energy-saving, and sustainable methods of titanium production, the molten salt electrolysis method holds the most promise [17,21]. Other research has provided a detailed description of the molten salt electrolysis process for titanium production, identifying the FFC, OS, and USTB methods as representative titanium production processes [8,22]. In addition, there are studies that have summarized the research progress on the extraction of titanium from TiO_2_ by electrolysis, pointing out that since the proposal of the FFC method, research on the direct electrolysis of oxides to produce titanium has become a hot topic [23,24]. Based on the summary of previous reviews [24,25], this study not only covers the main preparation processes of electrolytic titanium and their research progress but also delves into aspects such as process parameter optimization, research on the oxygen removal process and mechanism, and the improvement of electrolysis cells. More importantly, focusing on the main issue of the long and complex oxygen removal reaction path that hinders the application and the promotion of electrolytic titanium technology, this study examines this issue as the main thread. Starting from the long reaction paths of the FFC and OS methods, this study discusses the short path of the SHS-ED method and reviews the latest developments that can promote the application and popularization of electrolytic titanium technology.

In this review, we employed a systematic literature review methodology to ensure the comprehensiveness and rigor of our study. We defined a set of keywords in Web of Science, including “electrolytic titanium production”, “FFC Cambridge process”, “OS method”, “SHS-ED technique”, “USTB method”, “titanium powder production”, “electrolysis mechanism”, and “process optimization”, to search for relevant literature. We focused on articles published within the last 15 years to ensure that our review reflects the most recent advancements in the field. By analyzing current research findings and existing challenges, this article guides future research directions and illustrates the development of electrolytic titanium technology to achieve more efficient and environmentally friendly titanium production.

## 2. Historical Evolution of Early Electrolytic Titanium Technology

As early as 1904, Borchers and Hupperts reported on the electrolytic production of titanium in alkaline earth halide molten salts [24]. Electrochemical technology was considered a more economical and environmentally friendly titanium extraction technology compared to the Kroll process, and Kroll himself predicted that electrolytic technology would inevitably replace the Kroll process as the new method for producing metallic titanium [26,27]. Wainer et al. [28] first reported the production of metallic titanium by electrolyzing TiC-TiO solid solutions in 1955, which expanded the selection of precursors for the electrochemical production of metallic titanium and was of great significance to the development of the titanium industry. In 1960, the National Lead Company developed a method for the electrochemical reduction of titanium tetrachloride to metallic titanium using a cathode basket in a chloride molten salt [29]. Since then, the preparation of metallic titanium through electrochemical techniques using fluoride or chloride molten salts has been widely studied.

In 1967, the U.S. Bureau of Mines proposed a two-step reduction of TiCl_4_ to prepare metallic titanium, which involved the initial electrochemical reduction of TiCl_4_ to TiCl_2_, followed by the cathodic reduction of Ti^2+^ to metallic titanium. In 1981, Chassaing et al. studied the electrochemical deposition behavior of TiCl_3_ in three molten salts (LiCl, CsCl, and LiCl-KCl) at 700 °C [30]. Their research indicated that the reduction of Ti^3+^ is a two-step process: Ti^3+^ is first reduced to Ti^2+^ at −1.6 V relative to the reference electrode and then to metallic titanium at −2.1 V. In 1987, Chen et al. studied the electrochemical reaction of K_2_TiF_6_ in NaCl-KCl melt at 700 °C, and their results indicated that the reduction process of Ti^4+^ is a three-step reduction (Ti^4+^ → Ti^3+^ → Ti^2+^ → Ti^0^) [31]. In 1988, Ferry et al. used pulse techniques and alternating current impedance measurements to study the electrochemical reaction of Ti^2+^ in LiCl-KCl eutectic melt, and their results showed that the reduction of Ti^3+^ to Ti^2+^ is a quasi-reversible reaction, while the reduction of Ti^2+^ to Ti is irreversible [32].

Concurrently, electrochemical reactions in fluoride melts have also been extensively studied. In 1963, Stetson successfully prepared metallic titanium at the cathode using a NaF-KF-K_2_TiF_6_ mixed molten salt at 830 °C. In 1973, Clayton et al. utilized electrochemical testing techniques to study the cathodic electro-deposition behavior of K_2_TiF_6_ at 500 °C [33]. The results indicated that the cathodic reduction process of K_2_TiF_6_ in the mixed molten salt is a two-step reversible process. This process involves the initial reduction to Ti^3+^ at a relatively low negative potential, followed by the reduction of Ti^3+^ to metallic titanium at a relatively high negative potential. The reaction equations are shown in Equations (1) and (2). In 1986, Lepinayin et al. studied the cathodic electro-deposition behavior of K_2_TiF_6_ in LiF-KF and LiF-NaF-KF mixed molten salts at temperatures between 550 and 850 °C, arriving at similar conclusions [34]. In 1987, Ginatta reported a method for producing molten titanium by electrolyzing titanium tetrachloride in a mixed fluoride–chloride molten salt. Titanium tetrachloride was first dissolved in a CaF_2_-KCl molten salt to produce TiKCaF_6_ and CaCl_2_, followed by the electrolytic preparation of molten titanium, as shown in Equations (3) and (4)

(1) $${TiF}_{6}^{2-}+e^{-}\to {TiF}_{6}^{3-}$$

(2) $${TiF}_{6}^{3-}+{3e}^{-}\to Ti+6F^{-}$$

(3) $${3CaF}_{2}+KCl+{TiCl}_{4}\to {TiKCaF}_{6}+2{CaCl}_{2}+1/2{Cl}_{2}$$

(4) $${TiKCaF}_{6}+2{CaCl}_{2}\to Ti+{3CaF}_{6}+KCl+3/2{Cl}_{2}$$

In 1991, Robin et al. [35,36,37] determined the diffusion coefficients of TiF_6_^3−^ in LiF-NaF-KF mixed molten salts at 600 to 700 °C and revealed the interaction patterns between TiF_6_^3−^ and the cathode material during the electrochemical reduction process. This process could be coupled with the extraction of metallic titanium and the preparation of alloys, thus providing new insights for the production of titanium alloys. In 1999, Toshihide demonstrated the viability of electrolytic deposition of liquid titanium from a molten CaO-CaF_2_-TiO_2_ flux using Direct Current Electroslag Remelting (DC-ESR) technology [38]. The experimental findings revealed that by fine-tuning the electrode gap, the current efficiency could be optimized to 18%, and the deposition of pure titanium metal sheets was facilitated at greater electrode separations. Thermodynamically, the ample heat supplied within the furnace ensured the theoretical plausibility of the process, establishing a robust theoretical basis for the advancement and refinement of electrolytic deposition methodologies.

**Figure 1:**
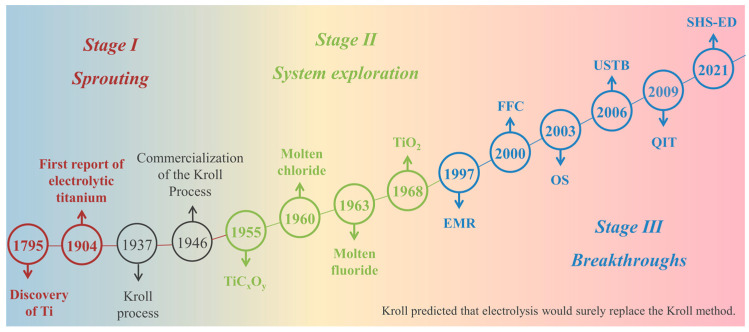
Development of electrolytic extraction technology for titanium.

Following in-depth research and analysis, we can divide the development of electrolytic titanium into three stages: the germination of methods, the exploration of systems, and the breakthrough of methods (as shown in Figure 1). Before 1950, with the discovery of titanium and the commercialization of the Kroll process, electrolytic titanium showed a germinative development since its first report in 1904. From 1950 to 2000, experts in the field of titanium metallurgy were committed to developing cost-effective electrochemical methods for extracting metallic titanium, which coincided with the concept of green chemistry. In 1968, Oki and Inoue [39] first directly electrochemically reduced titanium dioxide in molten salts, which was regarded as pioneering in the field of molten salt electroreduction of titanium dioxide and has since promoted the development of short-process reduction technology for metallic titanium. In the following chapters, we will introduce the key research progress in the stage of method breakthrough in recent years from the perspective of promoting the application and popularization of electrolytic titanium technology. 

## 3. Research Progress in Preparation of Titanium by Electrolysis

### 3.1. FFC Method

#### 3.1.1. FFC Process Overview

**Figure 2:**
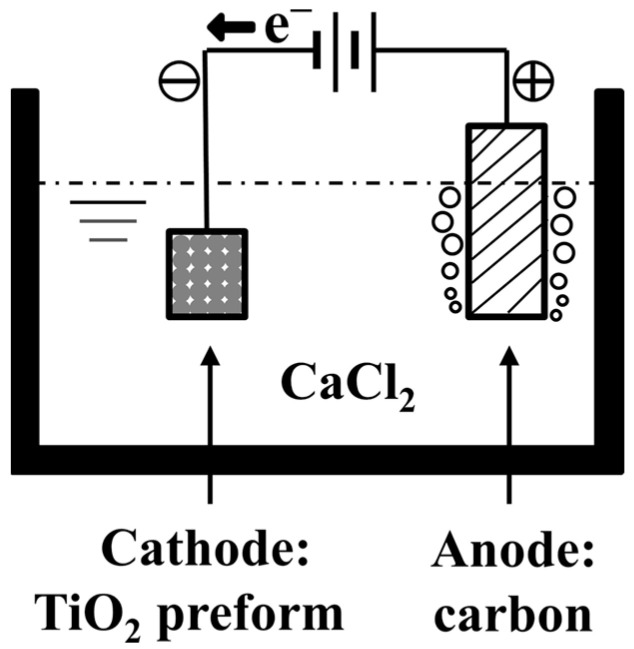
Schematic diagram of the FFC process.

In the year 2000, Fray et al. [40] proposed a novel process for the electrochemical conversion of titanium dioxide to metallic titanium in a molten electrolyte. The fundamental principle of the FFC Cambridge process involves the use of pre-sintered titanium dioxide as the cathode, molten calcium chloride as the electrolyte, and graphite as the anode [41]. During the process, oxygen within the solid titanium dioxide is ionized from the titanium dioxide under the influence of an external electric field and released at the anode, resulting in the deposition of metallic titanium at the cathode [42,43]. A schematic diagram of the process is shown in Figure 2. The cathode and anode reactions are as follows:

Anode: 2C + 2*x*O^2−^ = 2CO*_x_* + 4*x*e^−^ (5)

Cathode: TiO_2_ + 4e^−^ = Ti + 2O^2−^ (6)

Compared to the Kroll process, the key advantage of the FFC process is its ability to produce metallic titanium directly from titanium oxides without the need for traditional high-temperature reduction or chemical reduction steps, thereby simplifying the production process and potentially reducing costs [44,45]. However, the preparation of metallic titanium by this process typically requires extended over-electrolysis, leading to an overall low current efficiency. Preparing titanium powder with lower oxygen content in a shorter reaction time is the biggest challenge for this process to potentially replace the Kroll method [46,47]. The efficiency of the electrolysis process and the quality of the product are influenced by various factors, including electrolysis temperature, current density, and the composition of the electrolyte [48,49,50]. Studying the thermodynamics and kinetics of the transformation of TiO_2_ into Ti in the electrolyte helps us understand the entire electrochemical reaction process, thereby achieving the goal of adjusting process parameters to increase the reaction rate.

#### 3.1.2. FFC Advances in Electrolysis Mechanism

**Figure 3:**
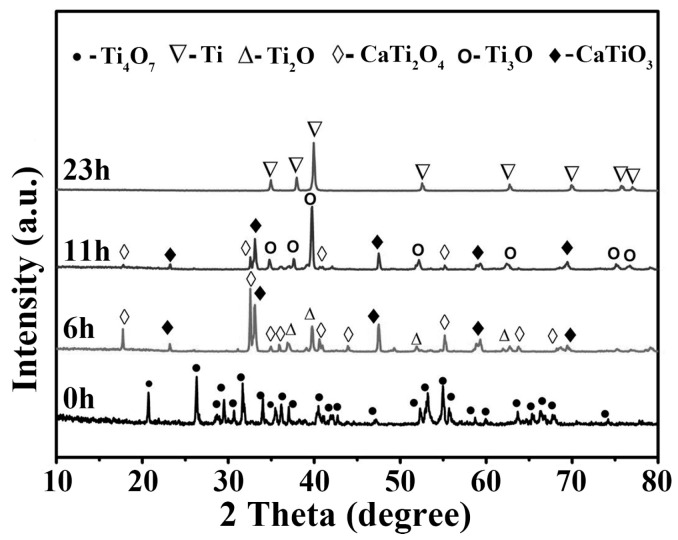
The XRD patterns of samples reduced at 900 °C for various times (0 h, 6 h, 11 h, 23 h) [53].

Schwandt et al. [51] investigated the thermodynamic process of the transformation of cathodic TiO_2_ into metallic titanium in CaCl_2_ molten salt. They believed that during the multi-step reduction of TiO_2_ to Ti in molten calcium chloride, the perovskite phase is an inevitable intermediate formed preferentially in the cathode during the early stages of electrochemical oxygen removal from TiO_2_. Although the perovskite phase can be further reduced to Ti under an electrochemical driving force, it also delays the electro-reduction kinetically. Lai [52] studied the initial reactions of forming CaTiO_3_ at the cathode and releasing Cl_2_ at the anode. The study found that at the beginning of the electrolysis, a large number of Ca^2+^ participate in the cathode reaction, leading to the accumulation of Cl^−^ at the anode and its release as Cl_2_. As the electrolysis process proceeded, the current–time curve showed a turning point after which the release of Cl_2_ ceases, which is related to the continuous formation of CaTiO_3_ at the cathode. Yang et al. [53] were the first to use Ti_4_O_7_ as the cathode material for the preparation of metallic titanium, providing a new approach for the efficient production of titanium metal using high electrical conductivity raw materials. However, during the transformation of Ti_4_O_7_ to Ti metal, intermediate products such as CaTiO_3_ and CaTi_2_O_4_ were still produced (Figure 3). Bhagat et al. [54] further analyzed the phase changes during the electro-oxygen removal process of TiO_2_ using in situ synchrotron X-ray diffraction testing. The study indicated that the formation of the perovskite phase was related to sub-stoichiometric TiO_2_. They suggested that if low-valence metal oxides (such as Ti_2_O_3_ and TiO) were used as raw materials for electro-oxygen removal, the formation of calcium titanate might be avoided. Tongxiang et al. [55] conducted electro-oxygen removal experiments using TiO, Ti_3_O_5_, and Ti_2_O_3_ as raw materials. The results showed that no calcium titanate phase was formed during the electrolysis of TiO; however, when the cathode raw materials were Ti_3_O_5_ and Ti_2_O_3_, the formation of the calcium titanate phase was still inevitable. The study also found that perovskite is formed by the combination of Ca^2+^ from the molten CaCl_2_ with the cathode material, mainly through the electrochemical deoxygenation process after the voltage was applied between the electrodes. As the oxygen content in the cathode decreased, the amount of perovskite gradually decreased.

Since it is difficult to avoid the formation of perovskite in the electrolysis process of TiO_2_, Jiang et al. [56] proposed a method of using perovskite to assist in the electro-oxygen removal of titanium dioxide to prepare metallic titanium. Compared with the direct electro-oxygen removal of TiO_2_, this method has a faster reduction rate and higher current density under the same experimental conditions. Alexander et al. [57] found that when the open porosity of the TiO_2_ precursor was reduced to 11–12%, the amount of CaTiO_3_ formed was greatly reduced compared to the electro-oxygen removal using porous TiO_2_ as the precursor. This finding expanded the degree of freedom for the design of precursors and process optimization in the process of preparing metallic titanium from TiO_2_. Furthermore, Xu [58] studied the preparation of titanium metal by electro-oxygen removal using CaTiO_3_ as the precursor in molten CaCl_2_-NaCl salt, further investigating the reduction process of CaTiO_3_ to titanium. The study revealed a multi-step process for the reduction of perovskite to titanium, including the initial decalcification and deoxygenation of CaTiO_3_ to form Ti_2_O_3_, followed by further deoxygenation of Ti_2_O_3_ to form TiO and a small amount of Ti_2_O, and finally, these low-valence titanium oxides continued to deoxygenate to form a solid solution of oxygen dissolved in titanium (Ti[O]_б_), which slowly transformed into metallic titanium. The reduction process of CaTiO_3_ was CaTiO_3_ → Ti_2_O_3_ → TiO/Ti_2_O → Ti[O]_б_ → Ti.

#### 3.1.3. FFC Electrolytic Process Optimization

**Figure 4:**
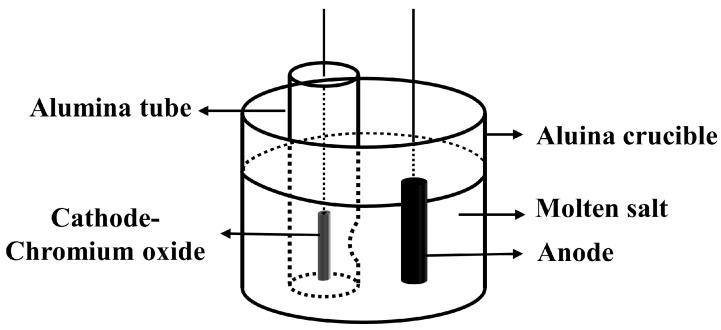
The schematic of the changed electrolytic cell.

Li et al. [59] experimentally verified the method of using NH_4_HCO_3_ as an additive to prepare high-porosity TiO_2_ precursors. By two-step voltage electrolysis (firstly electrolyzing at 3.2 V for 3 h and then continuing electrolysis at 2.6 V for another 3 h), they successfully prepared metallic titanium with an oxygen content of 0.19%, with energy consumption reduced to only 21.5 kWh/kg Ti, and the current efficiency was further improved to 32.3%. Mohanty and Behera [60] introduced a method for producing titanium metal by molten salt electrolysis using pre-treated TiO_2_ as the cathode material. The pre-treatment process involved mixing TiO_2_ with coal powder and ammonium fluoride, compressing and molding, and then thermally treating at 1000 °C for 1 h to partially reduce TiO_2_ to low-valence titanium suboxides. The results showed that pre-treated TiO_2_ can significantly reduce electrolysis time and improve the production efficiency of titanium metal, showing lower current consumption (23 kWh/kg) and higher current efficiency (31%). Hu et al. [61] studied the possibility of changing the design of the electrolysis cell to improve current efficiency. Figure 4 is a schematic diagram of the modified electrolysis cell. By placing the cathode in a perforated alumina tube, effective measures were taken to avoid short-circuiting and contamination of the cathode by carbon produced by the graphite anode. This modification significantly reduced the electric field strength inside the electrolysis cell, thereby greatly improving the current efficiency. In addition, this modification also reduced the issue of high background current while avoiding contamination of the cathode, reducing anode consumption, and greatly improving the deoxygenation effect of the sample.

Although graphite anodes perform well in some applications, they have limitations in fused chloride media, such as susceptibility to corrosion and CO_2_ production. Research into new anode materials is aimed at finding more stable and durable alternatives. Alzamani [62] used lanthanum-doped Ni_10_Cu_11_Fe_6_Al alloy as an inert anode to electrolyze titanium dioxide in molten calcium chloride at 900 °C and found that the sample doped with 0.0272 wt% lanthanum exhibited the best corrosion resistance after 16 h of electrolysis, with a corrosion depth of about 14 micrometers, and about 71 micrometers after 120 h of electrolysis, indicating good corrosion resistance in molten salts and can serve as an alternative to graphite electrodes. Lahiri and Jha [63] studied the efficient electrochemical reduction of titanium dioxide to metallic titanium by using an inert metal Al-Ti-Cu alloy as the anode instead of the traditional carbon anode. The use of an inert anode eliminated CO_2_ production at the anode, and under conditions with inert anodes and potassium ions, it promoted the decomposition of the perovskite phase, thus accelerating the electro-reduction kinetics, enabling the production of high-purity (with an oxygen content of only 1500 ppm) metallic titanium in just 16 h. Pertuiset [64] studied the electrochemical behavior of gold, palladium, and platinum as inert anode materials in the fused chloride electrolysis process and found that oxygen production could occur on the gold anode, but gold would also degrade due to the formation of AuCl_x_. Palladium required the formation of an oxide layer on its surface to produce oxygen, which was also destroyed by the formation of PdCl_x_. At an electrolysis current density of 0.03 A·cm^−2^ in a CaCl_2_-1 wt% CaO medium, the platinum anode was able to stably produce oxygen with minimal mass loss. In an article published in Nature, Akay [65] also pointed out that using inert, oxygen-producing electrodes for the FFC process to produce titanium is more valuable for space applications compared to using graphite electrodes (which produce CO and CO_2_).

### 3.2. OS Method

#### 3.2.1. OS Process Overview

**Figure 5:**
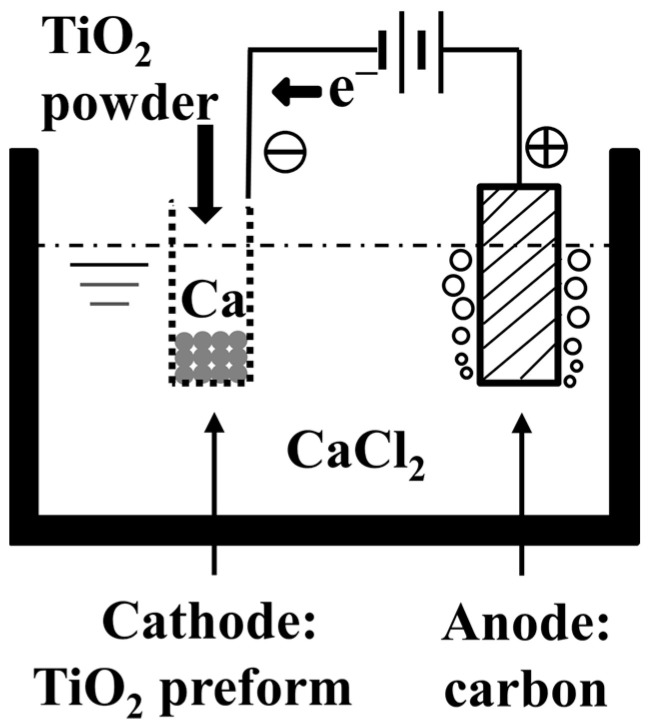
Schematic diagram of the OS process.

In 2003, Ono et al. proposed the electrolytic reduction of CaO in molten CaCl_2_ to produce metallic calcium for the direct cathodic reduction of TiO_2_, known as the OS process [66], as shown in Figure 5. In this process, the applied voltage should be higher than the decomposition potential of CaO and lower than the decomposition potential of CaCl_2_; metallic titanium with an oxygen content of 0.2% was prepared at 900 °C. The entire process can be described as the electrolytic reduction of TiO_2_ by Ca, which is produced from the cathodic electrolysis of an appropriate amount of CaO previously added in molten CaCl_2_, while the molten salt dissolves the CaO by-product, and the cathode regenerates the soluble reducing agent calcium through the dissolution of nearby CaO, ensuring the reducibility of the electrolyte [23,67]. Unlike the FFC method, the raw material used in this process is anatase-type TiO_2_ powder. The direct reduction of TiO_2_ at the cathode in the molten salt can be represented as:

Anode: 2C + 2*x*O^2−^ = 2CO_x_ + 4*x*e^−^ (7)

Cathode: 2Ca^2+^ + 4e^−^ = 2Ca (8)

Metallothermic reduction: TiO_2_ + 2Ca = Ti + 2O^2−^ + 2Ca^2+^ (9)

#### 3.2.2. OS Advances in Electrolysis Mechanism

Haraguchi et al. [68] conducted a detailed study on the side reactions during the preparation of metallic titanium by the OS method through cyclic voltammetry, especially the escape process of O^2−^ in molten salt. The results showed that O^2−^ first reacts with the graphite anode in the CaCl_2_ melt to form CO or CO_2_ bubbles, which disperse or dissolve in the molten electrolyte to form CO_3_^2−^, and finally, CO_3_^2−^ was reduced to produce carbon powder under reducing conditions. However, during the cathodic scanning process, no reduction peak of CO_3_^2−^ was observed, indicating that the production of carbon powder was related to the reduction of CO_3_^2−^ dissolved in the calcium chloride melt by dissolved metallic calcium. Shibuya et al. [69] studied the cathode morphology and thermal characteristics in a slightly hygroscopic LiCl-KCl-CaCl_2_ melt, and the measured heat absorption was significantly lower than the thermodynamic prediction, indicating that the generated hydrogen reacts with the liquid Ca deposited on the cathode surface to form hydrides. To solve the complexity and time-consuming nature of the TiO_2_ electro-deoxygenation process, Suzuki et al. [70] used SC_2_ to sulfidize TiO_2_ to prepare TiS_2_ as a precursor for the first time, which was successfully converted into metallic titanium with low residual sulfur content through the calcium thermal reduction and electrolytic reduction in the CaCl_2_-CaS melt. At a temperature of 1133 K in the calcium chloride melt, calcium was used as a reducing agent, and when the molar ratio of Ca/TiS_2_ was 2.0, the final Ti product had a sulfur residue of 0.03%. In the CaCl_2_-CaS melt, at a temperature of 1173 K, when the electrolytic reduction was carried out, and the charge quantity provided reached four times the theoretical required charge quantity, i.e., Q/Q_0_ = 4, the resulting Ti powder had a sulfur residue of 0.01%, but the residual oxygen content reached 0.96%.

#### 3.2.3. OS Electrolytic Process Optimization

**Figure 6:**
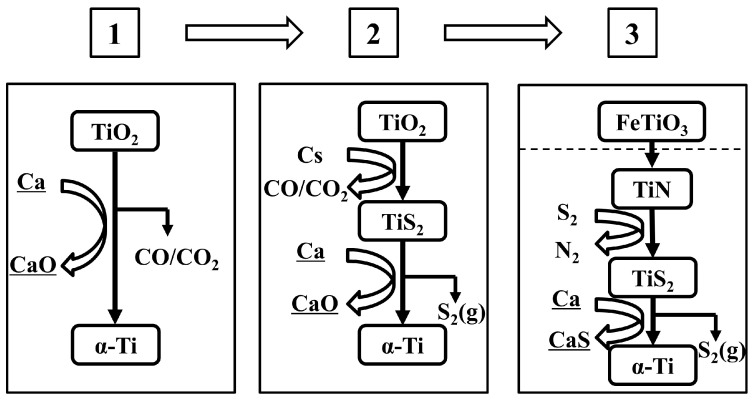
Progress of titanium powder prepared by OS process [71].

Figure 6 shows a schematic diagram of the optimization process of the titanium powder preparation process by the OS method in recent years, indicating the trend of precursor changes in the preparation of titanium powder by the OS method; that is, the precursor changes from TiO_2_ to TiS_x_, and the selection range of raw materials for preparing TiS_x_ gradually moves towards the upstream mineral resources, changing from TiO_2_ to TiC, TiN, and ilmenite.

**Figure 7:**
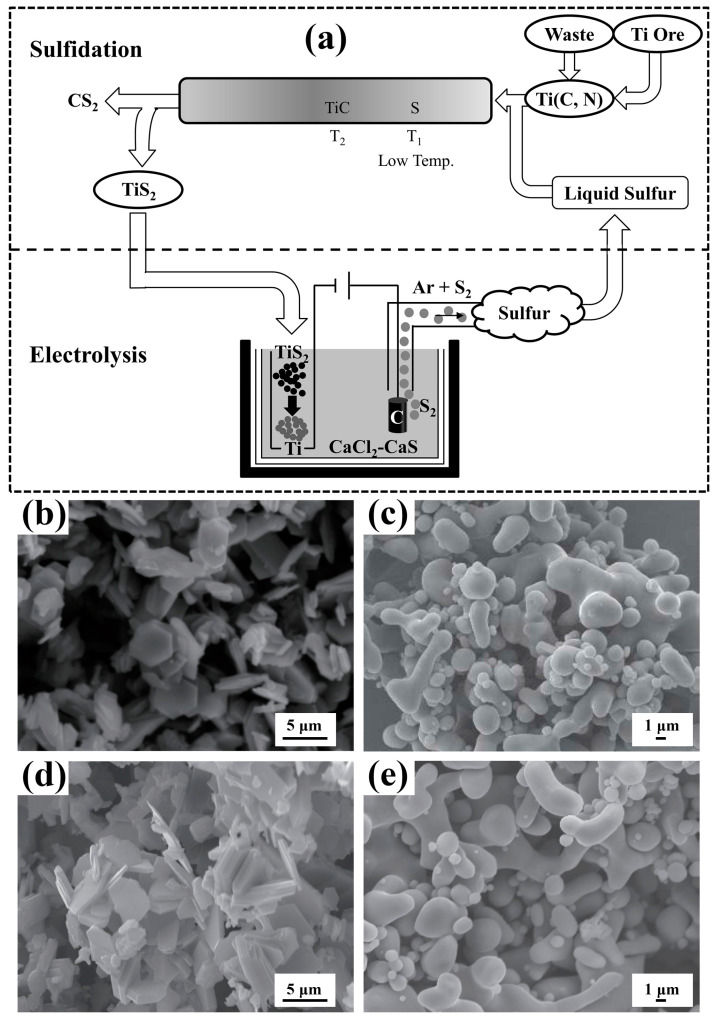
(**a**) Preparation of TiS_2_ and titanium powder by OS process; (**b**) TiS_2_ obtained by sulfidation of TiN; (**c**) Ti powder obtained by OS process from raw material (**b**); (**d**) TiS_2_ obtained by sulfidation of TiC; (**e**) Ti powder obtained by OS process from raw material in (**d**) [71].

Ahmadi et al. [72,73] transformed ilmenite into TiN or TiC and then into pure TiS for use as a precursor for the preparation of metallic titanium, using the in situ electrolysis of CaS to form the reducing agent Ca to desulfurize titanium and prepare metallic titanium. The process is shown in Figure 7a, and metallic titanium with low levels of sulfur, carbon, and oxygen residues has been successfully prepared, providing a new idea for the sustainable preparation of metallic titanium. At the same time, they studied the influence of process parameters on the final product quality, and the micro-morphology of the raw materials and products obtained is shown in Figure 7b–e. When the charge quantity Q/Q_0_ = 6, the final product obtained through FeTiO_3_ → TiN → TiS_2_ → Ti contained O < 0.15%, C < 0.02%, N < 0.003%, S < 0.002%. When the charge quantity Q/Q_0_ = 4, the final product obtained through FeTiO_3_ → TiC → TiS_2_ → Ti contained O < 0.125%, C < 0.02%, S < 0.005%.

Ono et al. [74] used 2 tons of molten salt as an electrolyte and conducted a 10-day experiment at a current of 4000 A, verifying the stability and feasibility of the process, but they did not describe the detailed experimental process or the information of the final product. At the same time, they proposed an industrial model for the preparation of metallic titanium by the OS method and put forward several requirements for the reactor needed in the actual titanium engineering production: (1) open reactor; (2) simple structural design; (3) permanent self-sustaining lining; and (4) surface protective layer on the electrolyte.

### 3.3. SHS-ED Method

#### 3.3.1. SHS-ED Process Overview

The innovation and breakthrough in the theory and key technology for the low-cost preparation of high-quality titanium powder/titanium alloy powder is the key to achieving the low-cost and large-scale application of titanium materials. Based on the deoxygenation limitation link of TiO_2_ (TiO → Ti) and the difference in affinity between titanium of different valences and oxygen, the Institute of Special Metallurgy and Process Engineering at Northeastern University proposed a new process for the preparation of low-oxygen titanium powder by self-propagating rapid reduction of TiO_2_ and electrochemical deoxygenation. Through this process, low-oxygen titanium powder with an oxygen content as low as 0.03% to 0.07% can be prepared, and a low-cost and clean preparation technology prototype for low-oxygen titanium powder (O < 0.1%) with independent intellectual property rights has been formed. First, TiO_2_ was subjected to self-propagating rapid reduction to obtain a non-stoichiometric, high-conductivity powdery low-valence titanium oxide TiO_x<1_ [75]; then, low-oxygen titanium powder was obtained by electrochemical deoxygenation of TiO_x<1_. This process has two significant advantages: (1) self-propagating rapid reduction can achieve low-cost and clean preparation of low-valence titanium oxide precursors without additional energy; (2) electrochemical deoxygenation, due to its technical characteristics, has a lower deoxygenation limit compared to traditional thermal reduction deoxygenation, which is conducive to the preparation of low-oxygen titanium powder.

#### 3.3.2. SHS-ED Research Progress

A series of low-valence titanium oxides were prepared by the metal titanium-TiO_2_ doping method, and the electrochemical deoxygenation process of low-valence titanium oxides was studied by cyclic voltammetry tests, I-t curves, phase analysis, and morphological observation, etc. It was shown that the cathodic electrochemical deoxygenation process of TiO and Ti_2_O does not generate CaTiO_3_. Under the electrolysis voltage of −3.3 V and 900 °C for 12 h, it was found that using low-valence titanium oxides with lower oxygen content can not only reduce the shrinkage rate and sintering strength of the final product, which is beneficial for obtaining metallic titanium in powder form, but also using TiO as the cathodic electro-deoxygenation precursor helps to avoid the formation of complex titanates. Therefore, it is confirmed that low-valence titanium oxides TiO_x<1_ as cathodic electro-deoxygenation precursors are beneficial to shortening the electro-deoxygenation path. TiO_x<1_ was prepared using magnesium thermal self-propagating and calcium thermal self-propagating methods, respectively.

**Figure 8:**
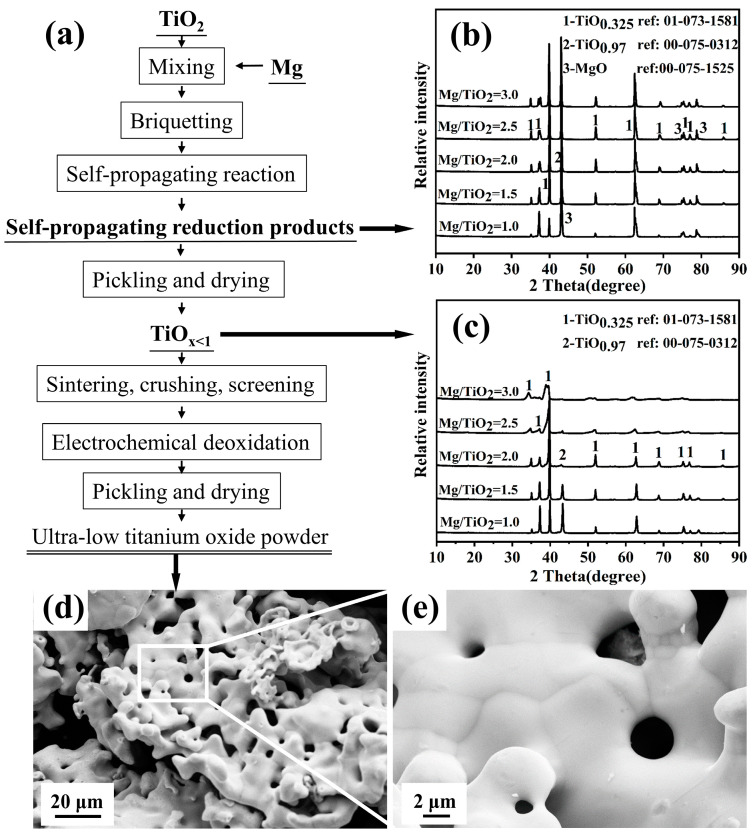
(**a**) Process flow chart for the preparation of low-oxygen titanium powder by magnesium thermal self-propagating electrolysis method. (**b**) XRD pattern of self-propagating rapid reduction product. (**c**) XRD pattern of the product after acid leaching. (**d**,**e**) Microstructure of the product.

The magnesium thermal self-propagating method successfully and rapidly reduced the preparation of TiO_x<1_, and the process flow chart is shown in Figure 8a. The XRD pattern of the self-propagating rapid reduction product and the XRD pattern of the product after acid leaching are shown in Figure 8b,c, respectively. With a raw material ratio of Mg/TiO_2_ = 1~3, the phase composition of the product prepared by magnesium thermal self-propagating rapid reduction-acid leaching is TiO_0.97_ and TiO_0.325_ of TiO_x<1_, and as the magnesium ratio increases, the proportion of TiO_0.325_ in the low-valence titanium oxide increases, and the median particle size of the powder gradually decreases. The lattice spacing of the low-valence titanium oxide is 2.9143 nm, in which the oxygen is present in the form of TiO_2_ and dissolved oxygen, and the thickness of the oxidation layer is less than 10 nm.

The electrochemical deoxygenation experiment was carried out using TiO_x<1_ prepared by magnesium thermal self-propagating as the precursor. The deoxygenation process, which includes two key steps of TiO → Ti(O) → Ti, was revealed by cyclic voltammetry experiments and chronoamperometry experiments. The electrolytic cell design and operating conditions were optimized, and the optimized process conditions were determined: a center-symmetric cathode basket (diameter 1~2 cm), a partitioned electrolytic cell, and an electrolysis voltage of −3.3 V at 900 °C. Through the process flow of sintering, crushing, screening, and electro-deoxygenation, low-oxygen titanium powder inheriting the morphology of the precursor was prepared, with a median particle size range of 96~250 μm and an oxygen content of 0.03%~0.06%. The researchers determined that a moderate particle size can avoid sintering during the electrolysis process, which is beneficial to the inheritance of morphology. A moderate sintering temperature can avoid sintering during the electrolysis process, and a larger specific surface area can provide more reaction sites for deoxidation.

**Figure 9:**
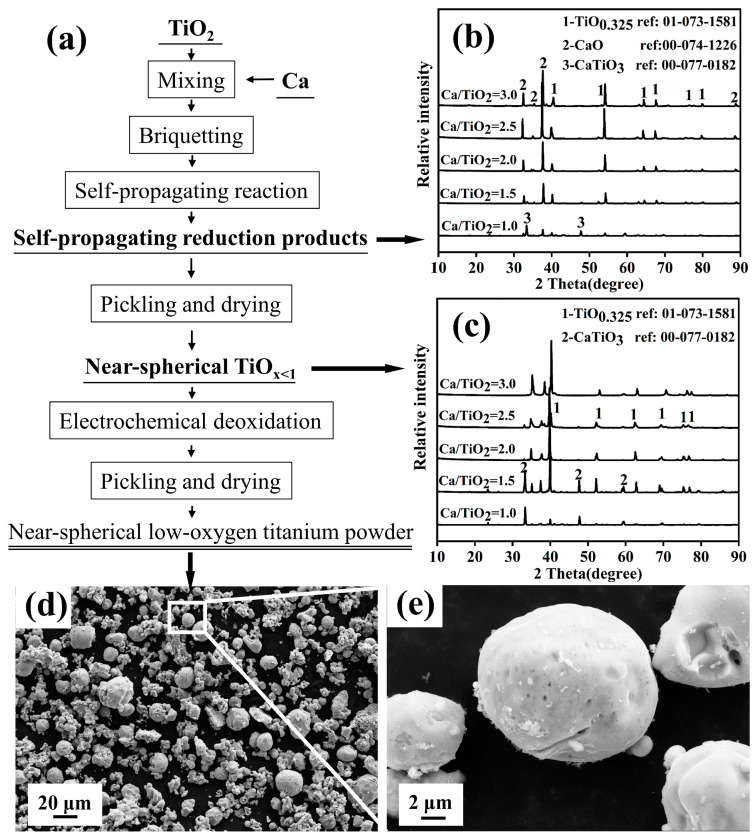
(**a**) Process flow chart for the preparation of nearly spherical low-oxygen titanium powder by calcium thermal self-propagating electrolysis method; (**b**) XRD pattern of self-propagating rapid reduction product; (**c**) XRD pattern of the product after acid leaching; (**d**,**e**) the microstructure of the product.

The calcium thermal self-propagating method successfully and rapidly reduced the preparation of nearly spherical TiO_x<1_. The process flow chart is shown in Figure 9a, and the XRD pattern of the self-propagating rapid reduction product and the XRD pattern of the product after acid leaching are shown in Figure 9b,c, respectively. The influence of process parameters, such as material ratio, pressing pressure, and reaction atmosphere, on the phase composition and morphological transformation of the Ca-TiO_2_ self-propagating reaction product was studied, and the optimized process parameters for the preparation of nearly spherical titanium powder were obtained: molar ratio Ca/TiO_2_ = 2.5~3, pressing pressure of 10 MPa, and reaction atmosphere pressure of 1~2 MPa. At the same time, the formation mechanism of nearly spherical TiO_x<1_ was studied. The morphological changes of low-valence titanium oxide during the Ca-TiO_2_ self-propagating rapid reduction process are jointly controlled by the reaction temperature, the forces during solidification, and the solidification medium. When the reaction temperature is close to or higher than the melting point of titanium metal (1668 °C), the liquid Ca medium containing titanium metal droplets tends to form a nearly spherical shape under the action of surface tension and the principle of minimum surface area during the rapid solidification process. Nearly spherical TiO_x<1_ underwent electro-deoxygenation to obtain nearly spherical titanium powder with an oxygen content as low as 0.45% to 0.9%, and the micro-morphology of the product is shown in Figure 9d,e. The reaction temperature of the self-propagating rapid reduction process and the amount of metallic calcium added play a decisive role in the micro-morphology of the reduced titanium powder.

### 3.4. Other Method

#### 3.4.1. USTB Method

The USTB process, developed by Zhu Hongmin and colleagues at the University of Science and Technology Beijing (USTB), is an innovative method for the preparation of metallic titanium using a soluble anode [76]. This process primarily consists of two stages: the fabrication of the anode material, a solid solution of TiO·*m*TiC (where 0 ≤ m ≤ 1), and the electrolytic production of metallic titanium in a molten salt medium.

The soluble anode is typically prepared through the carbothermal reduction of TiO_2_ [77] or by sintering a mixture of TiO_2_ and TiC [78]. This involves uniformly mixing TiO_2_ with carbon powder or TiC in a specific ratio, compacting the mixture into pellets under a pressure of 300 to 1000 kg·cm^−2^, and then sintering for 4 h under vacuum conditions at temperatures ranging from 1273 to 1673 K (1000 to 1400 °C) and less than 100 Pa. This process yields a soluble anode with good electrical conductivity.

The electrolysis stage employs the sintered solid solution as the anode, a steel rod as the cathode, and a mixture of NaCl/KCl as the electrolyte. Metallic titanium is produced through electrolysis at a temperature of 1073 K (800 °C). The electrochemical reactions occurring during the electrolysis process are as follows:

(10) $$Anode:TiC_{x}O_{y}-ne^{-}\to {Ti}^{n+}+CO+{CO}_{2}$$

(11) $$Cathode:{Ti}^{n+}+ne^{-}\to Ti(n<4)$$

As research progressed, the USTB method and its related technologies continuously achieved new advancements, with a significant accomplishment being the use of titanium nitride (TiN) as a consumable anode in the electrorefining process. In the study by Wang et al., the electrochemical dissolution of TiN as a consumable anode in NaCl-KCl molten salt was thoroughly investigated [79]. The study found that during the electrolysis process, the TiN anode produced nitrogen gas (N_2_), and the titanium ion species varied between Ti^2+^ and Ti^3+^, depending on the electrochemical dissolution potential of TiN. This discovery provided a new perspective for the USTB method, suggesting that using TiN as an anode material could achieve a more efficient and environmentally friendly titanium production process. Compared to the use of TiC_0.5_O_0.5_ anodes, the use of TiN reduced the waste that might be generated in the traditional USTB method, decreasing environmental pollution, and at the same time, the titanium metal obtained at the cathode through the TiN electrorefining process had a higher purity, with significantly reduced oxygen and nitrogen content.

**Figure 10:**
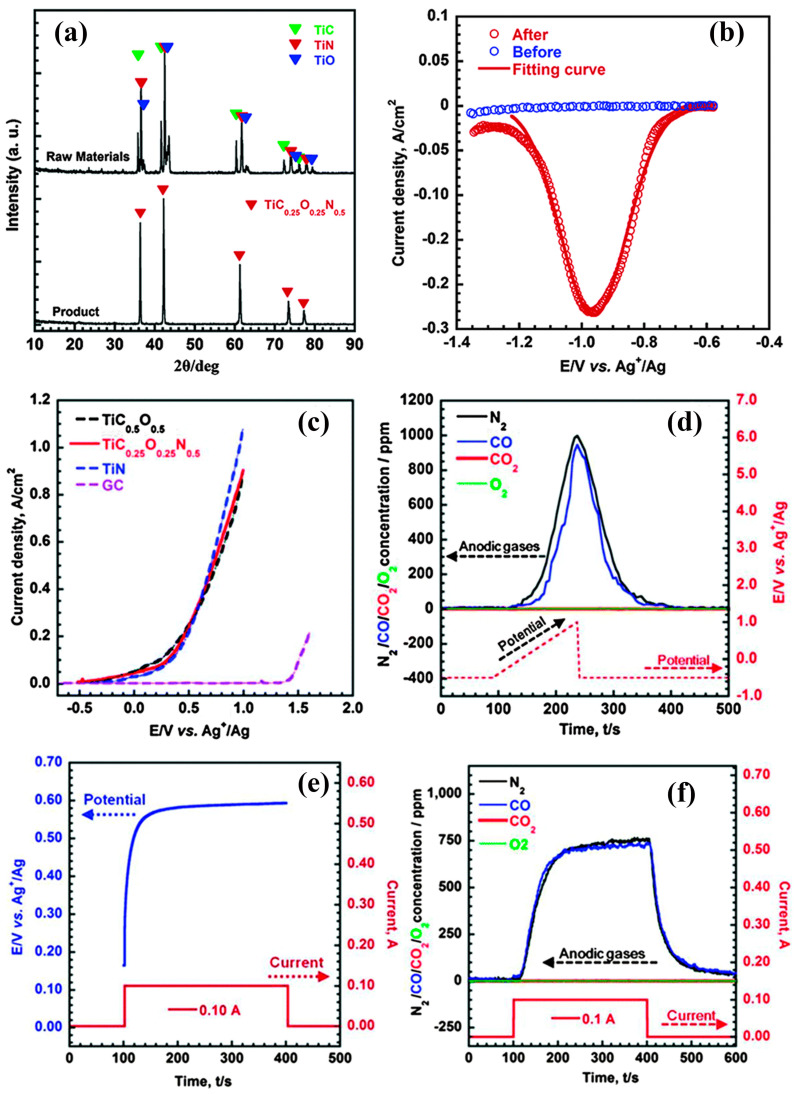
(**a**) The XRD patterns of the starting materials and products synthesized by spark plasma sintering at 1873 K. (**b**) Square-wave voltammetry of titanium from the TiO_0.25_C_0.25_N_0.5_ anode in the NaCl–KCl melts after electrolysis at a scan rate 50 mV s^−1^. (**c**) The anodic polarization curves of TiN, TiC_0.5_O_0.5_, TiO_0.25_C_0.25_N_0.5_, and GC electrodes at a scan rate 10 mV s^−1^. (**d**) Online gas analysis results of the TiO_0.25_C_0.25_N_0.5_ polarization experiment. (**e**) The potential–time plot of TiO_0.25_C_0.25_N_0.5_ anodic galvanostatic electrolysis in NaCl–KCl melts. (**f**) The profiles of the anodic gas concentration of N_2_, CO, CO_2_, and O_2_ during galvanostatic electrolysis [80].

Wang et al. successfully synthesized a novel single-phase titanium oxycarbo-nitride (TiC_0.25_O_0.25_N_0.5_) for the USTB titanium extraction process [80]. This material was synthesized by mixing TiO, TiC, and TiN in a 1:1:2 molar ratio and consolidating the mixture using Spark Plasma Sintering (SPS) technology at 1873 K. Figure 10a presents the XRD pattern of TiC_0.25_O_0.25_N_0.5_, confirming the crystal structure and purity of the new anode material. The square wave voltammetry in Figure 10b details the reduction behavior of titanium ions in the molten salt after dissolution from the TiO_0.25_C_0.25_N_0.5_ anode, confirming the generation of Ti^2+^ and further validating the redox mechanism of titanium during electrolysis through the calculation of the electron transfer number (n = 2.05). Figure 10c reveals the electrochemical polarization behavior of the TiO_0.25_C_0.25_N_0.5_ anode in NaCl-KCl molten salt, showing similar electrochemical activity to TiN and TiC_0.5_O_0.5_. Meanwhile, the online gas analysis results in Figure 10d indicate the production of N_2_ and CO gases during the anode dissolution process. Figure 10e,f further demonstrate the relationship between potential and time during constant potential electrolysis and the variation in anode gas concentration, thereby proving the stability and selectivity of TiO_0.25_C_0.25_N_0.5_ during the electrolysis process. Collectively, these results substantiate the effectiveness and superiority of TiC_0.25_O_0.25_N_0.5_ as a novel consumable anode material in the USTB process, offering a new solution for the low-cost, environmentally friendly extraction of titanium.

#### 3.4.2. MER Method

Il Park et al. investigated the production of titanium powder directly from titanium dioxide (TiO_2_) through an Electronically Mediated Reaction (EMR) using calcium as a reductant [81,82]. In their study, feed material (TiO_2_ powder or preform) and reductant (Ca–Ni alloy) were charged in electronically isolated locations in a molten calcium chloride (CaCl_2_) salt at 1173 K, and the current flow through an external circuit between the feed and reductant locations was monitored during the reduction of TiO_2_. The voltage between the feed electrode and reductant alloy was intermittently measured during the reduction experiment to monitor the reduction process. The results demonstrated that pure titanium powder with low nickel content could be obtained even though liquid Ca–Ni alloy was used as a reductant. In some experimental conditions, pure titanium powder with 99.5 mass% purity was successfully obtained. This approach offers a potential alternative to the traditional Kroll process for titanium production, potentially enabling the (semi-)continuous production of titanium powder, which is significant for improving the efficiency and purity of titanium manufacturing.

#### 3.4.3. QIT Method

The QIT (Quintuple Indirect Titanium) process is an electrochemical method for producing titanium or titanium alloys from liquid titanium mixed oxides, such as molten ilmenite, molten titanium slag, or molten TiO_2_. In this process, molten TiO_2_-containing material is used as the cathode and added to the reactor. A layer of molten salt or solid ion conductor is placed over the cathode as the electrolyte, and carbon is used as the consumable anode. Voltage is then applied to electrolyze the cathode and remove oxygen. The main reaction is as follows:

(12) $$TiO_{2}+C\to Ti+{CO}_{2}$$

During the electrolysis process, liquid titanium or titanium alloy was produced at the lower interface of the electrolyte and settled to the bottom of the reactor under the influence of gravity. At the same time, CO_2_ was produced at the anode. Since this process was initially developed for the extraction of titanium metal from titanium slag, the concentration of impurities, such as Fe, Mn, Cr, and Si, in the precursor was relatively high. Pre-treatment was necessary to remove these impurities and improve the purity and performance of the final product. The purity and quality of the product were influenced by various factors during the electrolysis process, such as the purity of the electrolyte, reaction temperature, and current density. By precisely controlling these parameters, the purity and performance of the titanium or titanium alloy were enhanced.

Zhao et al. introduced a novel short-process technology for the production of metallic titanium through the electrochemical extraction of carbon-doped titanium dioxide precursors in a chloride molten salt [83]. The experiments were conducted at 1123 K using carbon-doped TiO_2_ precursors. The electrochemical process of the carbon-chlorination intermediate products of carbon-doped TiO_2_ precursors was investigated. The results showed that by electrolyzing for 5 h at 4.0 V using original particles with a C/TiO_2_ mass ratio of 0.15, titanium powder with a purity of 98.7% could be obtained. In addition, low-valent titanium chlorides (TiCl_3_) as intermediate products have a higher production advantage. The Ti^3+^ ions are reduced to metallic titanium through a two-step mechanism, corresponding to the following pathway: Ti^3+^ → Ti^2+^ → Ti.

## 4. Process Comparison and Future Strategy

Table 1 shows a comparative analysis of the process parameters of titanium preparation by the electrolytic method, which integrates various research methods and results from the past ten years.

The progress in the FFC method primarily focuses on the impact of different anode materials, such as Al-Ti-Cu alloys, inert metal oxides, gold, palladium, platinum, etc., on the titanium powder production process. Additionally, efforts have been made to optimize reaction conditions, including temperature, voltage, and electrolytic cell design, to enhance the purity and production efficiency of titanium powder [88,89,90]. The use of carbon-based anodes in the FFC process leads to the generation of carbon dioxide, which not only reduces current efficiency but may also contaminate the cathode product through the formation of carbides. Therefore, seeking alternative inert anode materials to improve the sustainability of the process is a significant research direction for the future. Concurrently, the FFC process has relatively low current efficiency, necessitating a deeper understanding of the control of electrolysis conditions to increase current efficiency and reduce energy consumption.

The progress in the OS method mainly involves the study of titanium powder production using different electrolytes, such as CaCl_2_-0.5 mol pct CaS and CaCl_2_-TiCl_2_ molten salts, and different precursors, including TiC, TiN, and ilmenite. The research emphasis is on controlling the electrolysis process by adjusting electrolytic parameters and molten salt composition, as well as exploring new open-style reactor designs to improve the purity and production efficiency of titanium powder [91]. The future directions for the OS method are twofold: first, to enhance the efficiency of the electrolysis process and the purity of titanium by optimizing cell design, improving electrode materials, and refining electrolytic conditions; second, to facilitate the transition of the OS method from laboratory scale to industrial scale, achieving mass production to meet the market demand for titanium materials.

The SHS-ED method has successfully produced low-oxygen titanium powder and near-spherical titanium powder, achieving phased results that fully demonstrate and confirm the advantages of this method. However, a comprehensive theoretical system for this method has not yet been established. Based on the multifaceted theories, methods, and technologies involved in the SHS-ED process, there are still new challenges to address in the future. These include the development of cost-effective high-conductivity low-valent titanium oxide precursor production processes, more precise electrochemical sensing technologies for real-time monitoring of the electro-deoxidation process and determination of the endpoint, continuous electro-deoxidation processes, and the design of kilogram-scale electro-deoxidation devices.

The USTB process, which obtains metallic titanium from titanium ions electrochemically dissolved at the anode, avoids the issues of high oxygen content and the introduction of metal impurities common in the FFC and OS methods. The oxygen mass fraction in the electrolytic product titanium is less than 0.0003. Additionally, the use of highly conductive TiC_x_O_y_ as the anode significantly improves the current efficiency, with the laboratory current efficiency maintained at around 90%. This method can produce high-purity titanium with a titanium content greater than 99.9%, and the resulting product is also easy to separate. However, it is still in the industrial trial stage.

## 5. Conclusions

The value of titanium is increasingly recognized, making it a strategic metal essential for enhancing the quality and capabilities of defense equipment. The greening of titanium production processes is a crucial safeguard for the further development of the titanium industry. This review has provided a comprehensive overview of the latest advancements in electrolytic titanium production, focusing on key methods such as the FFC Cambridge process, the OS method, the SHS-ED technique, and the USTB method. These methods represent significant strides towards more efficient and environmentally friendly titanium production, addressing the limitations of traditional processes. Key advancements include the use of various anode materials in the FFC method, such as Al-Ti-Cu alloys and inert metal oxides, which improve the purity and efficiency of titanium powder production. The OS method has explored different electrolytes and precursors, optimizing the electrolysis process and reactor designs to enhance product purity and efficiency. The SHS-ED method, which has effectively produced low-oxygen and near-spherical titanium powder, boasts a significant advantage in its ability to shorten the deoxidation reaction path, thereby enhancing process efficiency and reducing the formation of undesirable phases, even though a more comprehensive theoretical framework is still needed. The USTB method, using highly conductive TiC_x_O_y_ anodes, achieves high current efficiency and produces high-purity titanium with low oxygen content. 

Despite these advancements, challenges remain. The FFC and OS methods require further optimization to improve current efficiency and reduce energy consumption. The FFC method’s use of carbon-based anodes leads to carbon dioxide generation, necessitating the search for alternative inert anode materials. The OS method needs to transition from laboratory to industrial scale, while the USTB method is still in the industrial trial stage.

These findings underscore the significance of ongoing research and development in electrolytic titanium production, which could reduce its environmental impact, enhance cost-effectiveness, and spur technological innovation. In summary, electrolytic methods are poised to play a pivotal role in the future of the titanium industry. Sustained research and development are essential to fully exploit the potential of these technologies, thereby ensuring the industry’s long-term sustainability and growth.

## Figures and Tables

**Table 1:** Comparative analysis of process parameters of titanium prepared by electrolytic method.

Method	Electrode	Electrolyte	Reaction Condition	Product Features	Process Characteristics	Reference
FFC	Al-Ti-Cu alloy anode	CaCl_2_-LiCl	900 °C, 3.1 V	Titanium powder (oxygen content 1500 ppm)	Reduced CO_2_ emissions, electrolysis time shortened to less than 16 h	[63]
FFC	Graphite anode, pre-treated TiO_2_ briquette cathode	CaCl_2_	1000 °C	Titanium powder	Pre-treatment of TiO_2_ reduced electrolysis time	[60]
FFC	Inert metal oxide anode	CaCl_2_	900 °C, 3.1 V	Titanium powder	Improved electrolysis cell, increased current efficiency	[61]
FFC	Gold, palladium, platinum inert anode	CaCl_2_-CaO (0–2 wt%)	850 °C	Titanium powder	Platinum electrode produced oxygen during electrolysis without easy damage	[64]
FFC	La-doped Ni10Cu11Fe6Al alloy inert anode	CaCl_2_	900 °C, 3.0 V	Titanium powder	Significantly improved anode corrosion resistance, reduced corrosion depth during electrolysis	[62]
FFC	Graphite anode, CaTiO_3_ cathode	CaCl_2_-NaCl	750 °C, 3.2 V,	Titanium powder	Direct preparation of titanium metal from CaTiO_3_, reducing intermediate steps	[58]
OS	Carbon rod anode	CaCl_2_-0.5 mol pct CaS	900 °C, 3 V	Ti powder, oxygen content 500–2000 ppm	Production of Ti and TiS_2_ in molten salt through sulfidation and electrolytic reduction of TiC	[84]
OS	Glassy carbon anode	CaCl_2_-TiCl_2_ melt	900 °C	High-purity titanium powder, oxygen content 240 ppm	Electrolysis using purified CaCl_2_ molten salt system to obtain high-purity titanium powder	[85]
OS	Carbon rod anode	CaCl_2_-CaO-Ca	870–900 °C, 3 V	Low-oxygen titanium powder	Designed a new open reactor, further discussion needed to determine anode design	[74]
OS	Carbon rod anode	CaCl_2_-0.5 mol% CaS	900 °C, 3 V	α-Ti powder, oxygen content below 0.15 wt pct	Production of commercial-grade Ti powder from FeTiO_3_ via TiS_x_ and TiN	[73]
OS	Carbon rod anode	CaCl_2_-0.5 mol% CaS	900 °C, 3.0 V	α-Ti, sulfur content 0.01 mass%	Electrolytic reduction of TiS_2_, low sulfur and oxygen content in metallic titanium	[70]
SHS-ED	High-purity graphite rod anode	CaCl_2_	900 °C, 3.3 V	Low-oxygen titanium powder, oxygen content 0.12%	Control of electrolysis time allows for regulation of titanium powder morphology and lattice parameters, providing possibilities for preparing titanium powder for specific uses	[86]
SHS-ED	High-purity graphite rod anode	CaCl_2_	900 °C, 3.0 V	Low-oxygen titanium powder, oxygen content 0.121%	Rapid preparation of TiO_x<1_ powder through self-propagating high-temperature synthesis, improving reaction speed and efficiency	[87]
USTB	TiO_0.25_C_0.25_N_0.5_	NaCl–KCl	750 °C	Pure titanium powders with N, O, C contents less than 300 ppm	Electrochemical dissolution of TiO_0.25_C_0.25_N_0.5_ anode, CO and N_2_ evolution	[80]
USTB	TiN	NaCl–KCl	750 °C	Titanium powder	Electrochemical dissolution of TiN anode, N_2_ evolution	[79]
USTB	TiC_x_O_1–x_	NaCl–KCl	750 °C	High-purity titanium metal	Electrochemical dissolution of TiC_x_O_1–x_ solid solutions, CO and CO_2_ gases generated	[77]

## Data Availability

No new data were created or analyzed in this study.
